# Plasma Nucleotide Dynamics during Exercise and Recovery in Highly Trained Athletes and Recreationally Active Individuals

**DOI:** 10.1155/2018/4081802

**Published:** 2018-10-09

**Authors:** Ewa A. Zarębska, Krzysztof Kusy, Ewa M. Słomińska, Łukasz Kruszyna, Jacek Zieliński

**Affiliations:** ^1^Department of Athletics, Strength and Conditioning, Poznan University of Physical Education, Królowej Jadwigi 27/39, 61-871 Poznań, Poland; ^2^Department of Biochemistry, Medical University of Gdansk, Dębinki 1, 80-211 Gdańsk, Poland; ^3^Department of General and Vascular Surgery, Poznan University of Medical Sciences, Długa 1/2, 61-848 Poznań, Poland

## Abstract

Circulating plasma ATP is able to regulate local skeletal muscle blood flow and 0_2_ delivery causing considerable vasodilatation during exercise. We hypothesized that sport specialization and specific long-term training stimuli have an impact on venous plasma [ATP] and other nucleotides concentration. Four athletic groups consisting of sprinters (n=11; age range 21–30 yr), endurance-trained athletes (n=16; age range 18–31 yr), futsal players (n=14; age range 18–30 yr), and recreationally active individuals (n=12; age range 22–33 yr) were studied. Venous blood samples were collected at rest, during an incremental treadmill test, and during recovery. Baseline [ATP] was 759±80 nmol·l^−1^ in competitive athletes and 680±73 nmol·l^−1^ in controls and increased during exercise by ~61% in competitive athletes and by ~31% in recreationally active participants. We demonstrated a rapid increase in plasma [ATP] at exercise intensities of 83–87% of VO_2_max in competitive athletes and 94% in controls. Concentrations reported after 30 minutes of recovery were distinct from those obtained preexercise in competitive athletes (*P* < 0.001) but not in controls (*P* = 0.61). We found a correlation between total-body skeletal muscle mass and resting and maximal plasma [ATP] in competitive athletes (r=0.81 and r=0.75, respectively). In conclusion, sport specialization is significantly related to plasma [ATP] at rest, during exercise, and during maximal effort. Intensified exercise-induced plasma [ATP] increases may contribute to more effective vessel dilatation during exercise in highly trained athletes than in recreational runners. The most rapid increase in ATP concentration was associated with the respiratory compensation point. No differences between groups of competitive athletes were observed during the recovery period suggesting a similar pattern of response after exercise. Total-body skeletal muscle mass is indirectly related to plasma [ATP] in highly trained athletes.

## 1. Introduction

The interest in skeletal muscle blood flow regulation has long and rich history but, recently, there has been a growing interest in this field, especially regarding exercise [1–7]. Several mechanisms controlling skeletal muscle blood flow were reviewed [8, 9]. Studies in this field focused on the contribution of purines and nitric oxide (NO) metabolites on vasodilatation. Additionally, adenosine has been proposed as a potent vasodilator and potential regulator of muscle blood flow [10, 11] although its role might not be as important in skeletal muscle substrate metabolism as its regulating blood flow properties [12, 13].

Optimizing O_2_ delivery to skeletal muscle is caused by release of both ATP and O_2_ from erythrocytes in regions of low O_2_ tension [14]. The released ATP binds to P2Y receptors on the endothelium and releases NO, endothelium-derived hyperpolarization factor (EDHF), and prostacyclins. As a result, local dilatation occurs, leading to increased blood flow to regions supplied by the vessel [2, 4].

ATP, in addition to functioning as an intracellular energy source, is equally important as an extracellular signalling molecule matching oxygen delivery with demand under physiological stress such as exercise [2]. In the skeletal muscle interstitium, there is a marked increase in ATP concentration, tightly coupled with the increase in blood flow. Skeletal muscle and endothelial cells are thought to be possible sources of interstitial ATP [5, 15]. ATP is also likely derived from red blood cells as they become deoxygenated and mechanically deformed when passing through the microcirculation of contracting skeletal muscle [16]. During exercise, the interstitial ATP concentration increases in direct proportion to the workload [17]. Moreover, a connection was noticed between exercise hyperaemia and an increase in venous [ATP], draining active muscle in proportion to exercise intensity [4, 18]. It was also noted that ATP increases in the feed artery and the vein that drains contracting muscle, which suggests that erythrocytes are the main origin of venous ATP [19]. ATP is released from the erythrocyte simultaneously with the offloading of O_2_ from the hemoglobin molecule. On the other hand, shear stress release of ATP from endothelial cells is suggested to be the main source of arterial ATP [4, 19, 20]. Notably, it has been shown* in vitro* that vasodilation of isolated resistance vessels in response to reduced O_2_ requires erythrocyte presence [2, 21].

During exercise, there is a dynamic interaction between vasoconstriction and vasodilation mediated by purine stimulation. Experimental studies performed on the vascular effects of ATP had crucial importance in revealing the complexities of this system [4, 22]. Extracellular ATP released from erythrocytes into the extracellular space in response to a variety of stress conditions acts as an important regulator of vascular homeostasis.* In vitro* data demonstrates that red blood cells release ATP when exposed to hypoxia in the presence of hypercapnia, hypoxia alone [4], and increased mechanical deformation [23]. Additionally, red blood cells release ATP in response to reductions in oxygen tension and pH [2, 4], elevated shear stress [24], and increased temperature [25]. These are typical conditions occurring during physical exercise, especially within the active skeletal muscle microcirculation. The physiological stimuli for* in vivo* ATP release in the vasculature of human muscle remain unclear, nonetheless recent data confirms that ATP release from erythrocytes is temperature dependent [26].

Nucleotide concentration plays a crucial role in adaptive processes during exercise [4]. However, there are still controversies concerning resting venous plasma ATP concentrations. Likewise, nucleotide concentrations during exercise still remain debatable. It has been shown that extracellular [ATP], [ADP], and [AMP] increase during submaximal and maximal intensity exercise [27]. González-Alonso et al. reported an increase in plasma ATP concentration during incremental knee-extensor exercise [4]. Other studies reported unchanged or slightly elevated plasma ATP concentrations during exercise [22, 28, 29]. Only a few studies showed changes in plasma ATP concentration during recovery after incremental [4, 19, 27] or exhaustive maximal exercise [20]. Cycle-ergometer or one-legged knee-extensor exercise protocol was used in those studies. To our knowledge, there are no studies showing changes in [ATP] during and after whole body exercise, e.g., treadmill exercise. Whole body exercise may provide additional information on changes in [ATP] that cannot be obtained using small muscle mass exercise protocols [4, 22, 29]. Moreover, there is no study concerning the effect of long-term training programs on plasma nucleotide concentration.

During aerobically dominant exercise, skeletal muscle blood flow increases to ensure appropriate supply of O_2_ sustaining the contractile activity of active skeletal muscle [30]. Skeletal muscle blood flow and oxygen delivery are strong predictors of aerobic exercise capacity [31]. During local maximal exercise, the skeletal muscle vascular bed is completely vasodilated [32] while during maximal whole body exercise, less active muscle fibers are under high vasoconstrictor influence of the sympathetic nervous system [30]. In trained humans during maximal whole body exercise, heart has insufficient capacity to supply oxygen and blood flow to exercising muscles [33]. Circulating ATP is able to regulate local skeletal muscle blood flow and O_2_ delivery by causing considerable vasodilatation and overcoming increased sympathetic vasoconstrictor activity [22]. Long-lasting physical training program can cause specific adaptations in response to specific demands of training type, especially muscle metabolism and O_2_ uptake. Previous studies have shown that among endurance-trained athletes an increased vasodilatation capacity during maximal exercise is mainly due to enhanced vasodilatory capacity [32, 34].

The main purpose of this study is to assess the effect of long-term training program on plasma nucleotide concentration and total-body skeletal muscle mass (ATP_SMM_) among highly trained athletes of different sport specializations in response to incremental treadmill test until exhaustion. We hypothesize that (1) training type has an impact on magnitude of ATP response during exercise and recovery among highly trained athletes and that (2) ATP efflux will depend on skeletal muscle mass.

## 2. Materials and Methods

### 2.1. Subjects

The study included 41 highly trained male athletes from different sport disciplines. The athletic groups consisted of sprinters (SP; n=11; age range, 21-30 yr), endurance-trained subjects including long-distance runners and triathletes (EN; n=16; age range, 18–31 yr), and futsal players (FU; n=14; age range, 18–30 yr). All athletes were members of the Polish national team. The control group consisted of 12 healthy recreationally active men aged 22–33 yr without previous and current competitive sport experience. The controls were invited through announcements via local mass media to participate in this study. More detailed characteristics of the study participants are presented in Table 1.

The project was approved by the Ethics Committee at the Karol Marcinkowski Poznan University of Medical Sciences and has been performed according to the ethical standards laid down in the Declaration of Helsinki. The participants were fully informed of the purpose and risks of the study before giving their written consent to participate. They were also recommended to avoid high-intensity and long-duration training sessions 24–48 h before the tests. All tests were conducted at the Human Movement Laboratory of the Poznan University of Physical Education at the end of the transition phase of the annual training cycle. The measurements were performed in the morning, 2 h after light breakfast (bread and butter, water, without coffee or tea). Prior to an exercise test, body composition assessment was performed. Then, subjects performed an incremental treadmill test until exhaustion. During all examinations, the ambient temperature remained unchanged at 20–21°C.

### 2.2. Body Composition Analysis

The subjects' body mass and height were measured using digital stadiometer (SECA 285, SECA, Hamburg, Germany). To evaluate body composition (total-body fat and appendicular lean soft tissue) the dual X-ray absorptiometry method (DXA, Lunar Prodigy; GE Lunar Healthcare, Madison, WI, USA) was used. Each day, prior to measurements, the DXA device was calibrated using a phantom, according to manufacturer guidelines. All DXA scans were performed and analyzed by the same trained technician according to manufacturer's protocols using enCORE 16 SP1 software. During the examination, subjects were wearing only their underwear, without any jewelry or metal objects to minimize measurement error. Total-body skeletal muscle mass (SMM) was calculated using Kim's et al. regression model [35].

### 2.3. Respiratory Parameters

All athletes underwent an incremental running treadmill test (H/P Cosmos Pulsar, Sports & Medical, Nussdorf-Traunstein, Germany) in order to determine maximal oxygen uptake (VO_2_max). The initial speed was set up at 4 km·h^−1^ and was increased after 3 min to 8 km·h^−1^. After that point, the moving strip was progressively increased by 2 km·h^−1^ every 3 min until an athlete reached voluntary exhaustion. After 10 km·h^−1^, blood samples were drawn at the end of each 3-minute stage. Main cardiorespiratory variables (minute ventilation, V_E_; oxygen uptake, VO_2_; carbon dioxide production, VCO_2_) were measured constantly (breath by breath) using MetaMax 3B-R2 ergospirometer and analyzed using MetaSoft Studio 5.1.0 Software (Cortex Biophysik, Leipzig, Germany). Maximal oxygen uptake was considered achieved if at least three of the following criteria were met: (i) a plateau in VO_2_ despite an increase in speed; (ii) cutoff blood lactate concentration ≥ 9 mmol·l^−1^; (iii) respiratory exchange ratio ≥ 1.10; and (iv) heart rate ≥ 95% of the age-predicted HR_max_ [36]. The respiratory compensation point (RCP) was determined from breath-by-breath data automatically at the inflection of the V_E_ versus VCO_2_ slope (V-slope method) [37]. A nonlinear increase in V_E_/VCO_2_ and a point where PETco_2_ begins to fall were determined when the V-slope method was insufficient and visual assessment was needed [38]. Before each test, the system was calibrated according to the manufacturer's instructions. Heart rate was measured continuously with Polar Bluetooth Smart H6 monitors (Polar Electro Oy, Kempele, Finland).

### 2.4. Blood Sampling

Venous blood samples were obtained at rest, at the end of each 3-min stage above 10 km·h^−1^, immediately after exercise and in the 5th, 10th, 15th, 20th, and 30th min of postexercise recovery. The catheter (1.3 × 32 mm, BD Venflon Pro, Becton Dickinson, Helsingborg, Sweden) was inserted retrogradely into the antecubital vein which was kept patent with isotonic saline (0.9% NaCl) during the whole procedure. Syringes comprising EDTA (S-Monovette, 2.7 ml KE, Sarstedt, Nümbrecht, Germany) were used for hematological parameters and plasma nucleotide concentrations analyses. For lactic acid measurement, lithium heparin as an anticoagulant (S-Monovette, 2.7 ml KE, Sarstedt, Nümbrecht, Germany) was used.

### 2.5. Lactic Acid and Hematological Measurements

Lactate in whole blood (20 *μ*l) was immediately assayed using the spectrophotometric enzymatic method (Biosen C-line, EKF Diagnostics, Barleben, Germany). 10 *μ*l of blood was used for hematological analysis carried out on an 18-parametric automated hematology analyzer Mythic®18 (Orphée, Geneva, Switzerland).

### 2.6. Plasma ATP, ADP, and AMP Measurements

For plasma nucleotide concentration analyses, 2 ml of blood was pipetted into a test vial (Eppendorf, Wesseling-Berzdorf, Germany) and immediately centrifuged (Universal 320R, Hettich Lab Technology, Tuttlingen, Germany) for 30 seconds at 14 000 rpm at 4°C. Then, plasma (200 *μ*l) was pipetted into 1.5 ml vials in duplicate and frozen down in liquid nitrogen. All samples were stored in -80°C until further analysis. Nucleotide degradation in blood is rapid; therefore the start of centrifugation was within 5 sec from collection, and plasma was deproteinized immediately after 30 sec centrifugation. The duration of the whole procedure from blood withdrawal to plasma separation did not exceed 45 seconds. The method of blood sampling for plasma ATP was tested for hemolysis that was absent.

### 2.7. Chromatography Method

Nucleotides in plasma were determined by high performance liquid chromatography (HPLC) with UV detection according to the methodology of Smolenski et al. [39] and Smolenski & Yacoub [40]. In brief, samples were extracted using perchloric acid (2.4 mol·l^−1^) on ice at the ratio of 1:0.25 for 15 min and then centrifuged at 13 000 rpm for 3 min at 4°C. Supernatant was collected and neutralized using 3 mol·l^−1^ K_3_PO_4_ before being centrifuged at 13 000 rpm for 3 min at 4°C.

The samples were left on ice for 30 min to ensure complete precipitation of potassium perchlorate. After that, supernatants were collected, transferred to another test tube, and stored at -80 before analysis. The analyses were performed using Spectra HPLC system (Thermo, USA) equipped with 10 cm path flow cell to increase sensitivity. Separation was achieved with analytical column BDS Hypersil C18 (150 mm x 4.6 mm x 3 *μ*m; Thermo, USA) placed in a thermostat (18°C) protected by precolumn 20 mm x 4 mm (Phenomenex, type SecurityGuard, USA). The mobile phase consisted of A: 122 mM KH_2_PO_4_, 150 mM KCL, and 28mM K_2_HPO_4_ and B: 15% (v/v) acetonitrile in A. The percentage of B changed from 0% to 100% in several linear steps during analysis and then returned to 0% B for reequilibration. The separation time with reequilibration was 13.5 min and was conducted at 0.9 ml/min flow rate. The sample injection volume was 40 *μ*l. The quantitative analyses were performed based on external calibration of the signal at 254 nm. Data acquisition and processing were managed by the Xcalibur™ software (v. 2.1, Thermo Scientific™, Waltham, MA, USA). The above described method provides good recoveries (>95%) and an acceptable coefficient of variation (<10%). Detection limit with high injection volume and 10 cm path flow cell in a detector used here was 50 nmol·l^−1^ for plasma ATP.

### 2.8. Statistical Analyses

To test changes in plasma ATP, ADP, and AMP concentration between measurement points during exercise and recovery within each group of participants, a one-way repeated-measures ANOVA was performed. We also used one-way repeated-measures ANOVA to test differences at the same measuring point between groups. If a significant difference was found (*P* < 0.05), post hoc Scheffe tests were conducted. The effect sizes for ANOVA analyses were small to very large for descriptive characteristics (*η*^2^ = 0.02–0.68). At the same measuring point, the effect sizes of ANOVA analyses between groups were very large for plasma ATP (*η*^2^ = 0.39–0.89), ATP_SMM_ (*η*^2^ = 0.15–0.68), ADP (*η*^2^ = 0.41–0.88), and AMP (*η*^2^ = 0.41–0.87). The effect sizes of ANOVA values were very large within groups between measurement points (*η*^2^ = 0.82–0.96). The statistical power of most observed ANOVA analyses for plasma ATP, ATP_SMM_, ADP, and AMP was 1.00. Only in the case of three variables, statistical power ranged from 0.66 to 0.97. Pearson correlation coefficients were used to describe the relationship between total-body skeletal muscle mass and plasma ATP concentrations at rest or maximal exercise. All statistical analyses were performed using STATISTICA 12.0 software (StatSoft, Tulsa, OK). Significance level was set at *P* < 0.05. All values were presented as means ± SD.

## 3. Results

### 3.1. Subjects Description

All athletes did not differ significantly with regard to height, body mass index (BMI), resting and maximal lactate concentrations, and maximal heart rate (Table 1). Sprinters had significantly smaller percent of total tissue fat than futsal players and controls; in addition, sprinters were characterized by significantly larger total-body skeletal muscle mass than compared groups. Competitive athletes had statistically similar training experience. Subjects from control group were engaged in endurance type recreational sports activities (3–5 times a week) but none were competitive athletes. Endurance athletes presented a significantly higher $\dot{V}O_{2}$max than others. In addition to that, VO_2_max per unit of total-body skeletal muscle mass was higher in futsal players and controls compared to sprinters. No significant differences in RBC, Hb, Hct, MCHC, and RDW (%) between compared groups were found. Sprinters had significantly lower MCV and MCH than endurance athletes who had also higher MCH values compared to futsal players.

### 3.2. Preexercise Nucleotide Concentration

**Figure 1:**
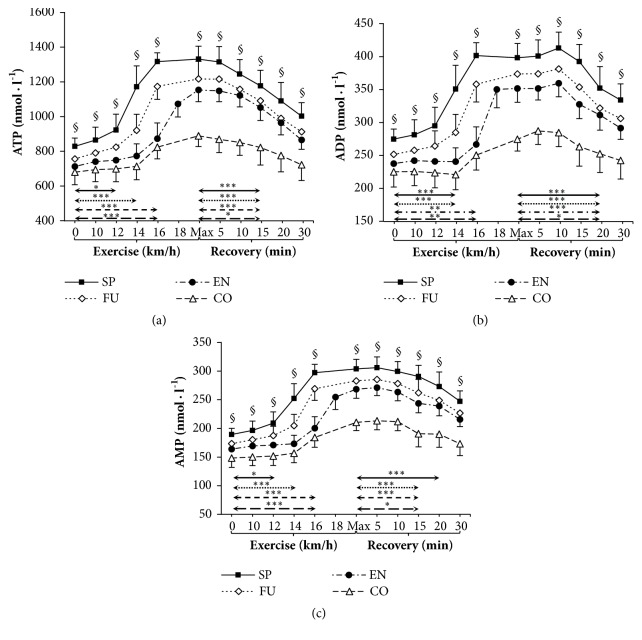
Venous plasma ATP, ADP, and AMP levels before exercise, during incremental treadmill test until exhaustion, and postexercise recovery in sprinters (n=11), futsal players (n=14), endurance athletes (n=16), and control group (n=12). Data are means ± SD. Arrows indicate the first significant differences from samples taken at rest and maximal exercise within groups. Significant differences between blood sampling points: *∗∗∗P* < 0.001, *∗∗P* < 0.01, *∗P* < 0.05. Significant differences between groups at the same measurement point: §*P* < 0.001.

Resting venous plasma [ATP], [ADP], and [AMP] differed between groups. Sprinters had significantly higher plasma [ATP], [ADP], and [AMP] than endurance athletes and controls (*P* < 0.001 both). Preexercise plasma [ADP] and plasma [AMP] were also significantly higher in futsal players than in control group (*P* < 0.05) (Figure 1).

### 3.3. Nucleotide Concentration during Exercise

Our data showed significant increases in plasma [ATP], [ADP], and [AMP] during exercise (Figure 1). During incremental exercise plasma [ATP] and [AMP] increased progressively, being significantly higher after 12 km·h^−1^ in sprinters (11.3% and 22.4% increase, respectively), 14 km·h^−1^ in futsal players (21.8% and 18.1% increase, respectively), and 16 km·h^−1^ in endurance athletes (22.5% and 22.4% increase, respectively) and in control group (21.2% and 21.2% increase, respectively) compared with rest (Figure 1). First significant increase in plasma [ADP] was observed after 14 km·h^−1^ in sprinters (27.6%) and futsal players (13.2%) compared to resting values. In the endurance and control groups, the first observed significant increase was after 16 km·h^−1^ (12.2% and 11.1%, respectively). It should be noted that only endurance athletes reached 18 km·h^−1^ during their test. Moreover, venous plasma [ATP], [ADP] and [AMP] at a given workload tended to be significantly higher in sprinters than endurance athletes and controls (*P* < 0.001 for v = 10 km·h^−1^) and all other groups beginning with 12 km·h^−1^ (*P* < 0.05 for FU; *P* < 0.001 for EN and CO), 14 km·h^−1^, and 16 km·h^−1^ (*P* < 0.001). All athletes reached their peak plasma [ATP] at the end of the test. The absolute average net increase in plasma [ATP] was 502 nmol·l^−1^ in sprinters (60.6%, 1.61-fold), 461 nmol·l^−1^ in futsal players (60.9%, 1.61-fold), 441 nmol·l^−1^ in endurance athletes (61.9%, 1.62-fold), and 209 nmol·l^−1^ in the control group (30.8%, 1.31-fold). None of the athletic groups reached peak plasma [ADP] and [AMP] at maximum intensity at the end of exercise.

### 3.4. Nucleotide Concentration during Recovery

During recovery there was a significant decrease in venous plasma nucleotide concentration in all groups. The first significant plasma [ATP] decrease compared to peak value was observed after 15 minutes from test completion in sprinters (11.5%), futsal players (10.4%), endurance athletes (11.5%), and control group (7.6%). The first observed significant plasma [ADP] decrease was observed after 20 minutes in sprinters (11.5%), futsal players (13.9%), endurance-trained group (11.4%), and controls (8.0%). The first significant decrease compared to peak value in plasma [AMP] was observed within 15 minutes of recovery in futsal players (7.4%), endurance athletes (9.1%), and control group (8.8%). In sprinters, the first significant plasma [AMP] decrease was detected after 20 minutes (10.2%). The absolute average net decrease of plasma [ATP] was 329 nmol·l^−1^ in sprinters (24.7%, 1.33-fold), 304 nmol·l^−1^ in futsal players (25.0%, 1.33-fold), 288 nmol·l^−1^ in endurance athletes (25.0%, 1.33-fold), and 167 nmol·l^−1^ in control group (18.8%, 1.23-fold). During recovery, venous plasma [ATP], [ADP], and [AMP] differed in all groups compared to controls (Figure 1.). Additionally, values reported after 30 minutes of recovery were distinct from those obtained preexercise in competitive athletes (*P* < 0.001), but not in controls (*P* = 0.61).

### 3.5. Changes in ATP Normalized to Skeletal Muscle Mass

**Figure 2:**
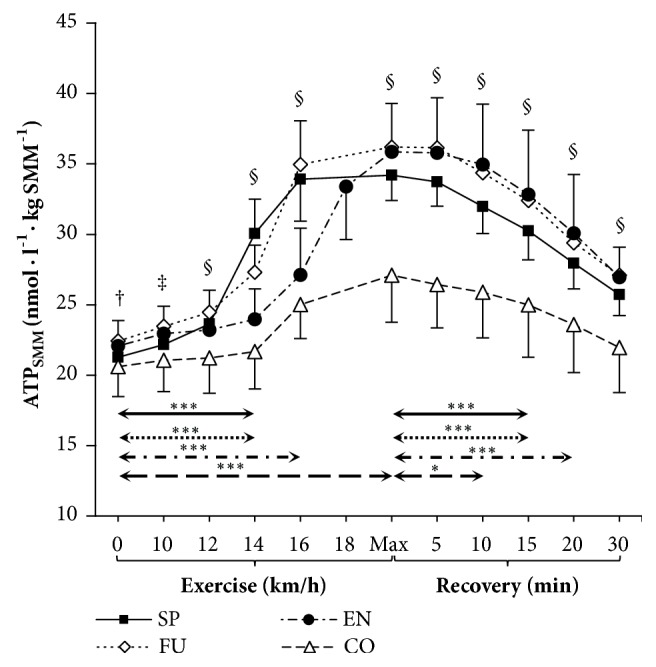
Venous plasma ATP concentration per kilogram of skeletal muscle mass (ATP_SMM_) before, during, and after incremental treadmill test until exhaustion in sprinters (n=11), futsal players (n=14), endurance athletes (n=16), and control group (n=12). Data are means ± SD. Arrows indicate the first significant differences from samples taken at rest and maximal exercise within groups. Significant differences between blood sampling points: *∗∗∗P* < 0.001, *∗P* < 0.05. Significant differences between groups at the same measurement point: §*P* < 0.001, ‡*P* < 0.01, †*P* < 0.05.

**Figure 3:**
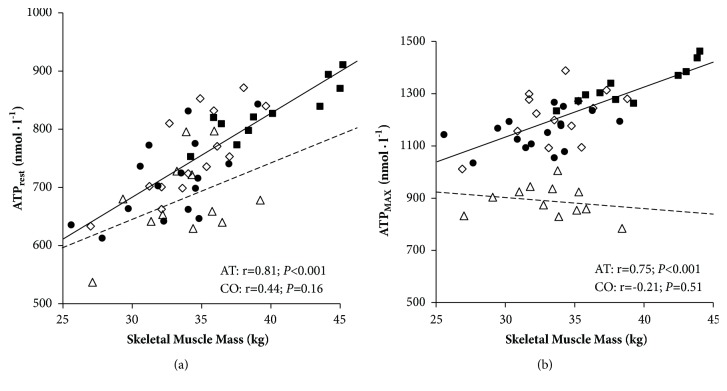
Relationship between skeletal muscle mass and plasma ATP concentration at rest (a) and maximal exercise (b) in competitive athletes (AT, solid line; including sprinters SP, n=11, ■; futsal players FU, n=14 ◊, and endurance athletes EN, n=16, ●) and control group (CO, n=12, dashed line, ∆).

Plasma [ATP] in relation to skeletal muscle mass before, during, and after exercise is expressed graphically in Figures 2 and 3. At rest, at peak exercise, and during the recovery period, venous plasma [ATP] per kilogram of skeletal muscle mass did not differ between groups of competitive athletes. During incremental exercise plasma [ATP_SMM_] increased progressively, being significantly higher after 14 km·h^−1^ in sprinters and futsal players, after 16 km·h^−1^ in endurance athletes, and immediately after maximal exercise in control group compared to rest (Figure 2). Resting and maximal plasma [ATP] was positively correlated with total-body skeletal muscle mass among competitive athletes, most strongly in sprinters (r=0.89; *P* < 0.001 both) (Figure 3) while no correlations were found in the control group.

### 3.6. Respiratory Compensation Point

Respiratory compensation point (RCP) expressed as a percent of $\dot{V}O_{2}$max occurred within 83–88% in competitive athletes and at 94% in control group. RCP occurred between 12 and 14 km·h^−1^ in futsal players, sprinters, and controls. Endurance athletes obtained RCP between 16 and 18 km·h^−1^. A rapid increase in plasma [ATP] occurred concomitantly with RCP.

## 4. Discussion

In this study, we analyzed venous plasma [ATP] in response to incremental exercise. The results showed that distinct sport specializations have different effects on plasma [ATP] in highly trained competitive athletes. However, no differences between the groups of competitive athletes were observed during recovery. Our study suggests that long-term training has an impact on plasma [ATP] responsiveness since recreationally active subjects showed significantly lower increments in response to exercise (~61% in competitive athletes versus ~31% in healthy recreationally active participants). These differences can be partly explained by vascular remodelling within muscle due to specific training adaptations resulting from sport type [41]. In highly trained athletes at similar sport level, absolute skeletal muscle mass seems to affect plasma [ATP], because after expressing [ATP] per kilogram of total-body skeletal muscle mass there were no differences between competitive athletes at rest, at maximal intensity, and during recovery. In our study, we confirmed a rapid increase in plasma [ATP] at exercise intensities of 83–87% of VO_2_max in competitive athletes and at 94% of VO_2_max in controls. We noticed that this sharp rise in plasma [ATP] is concomitant with the respiratory compensation point which reflects the partial inability to supply O_2_ to muscles during exercise.

Skeletal muscle blood flow regulation and oxygen delivery during exercise is complex and involves the mechanical effects of muscle contraction, presence of red blood cell and endothelium-derived substances, and sympathetic nervous system activity. Initially, this effect was attributed to the muscle pump effect, but it is now firmly accepted that the temporary increase in blood flow is mainly due to local vasodilatory response [42]. To date, there have been relatively few studies investigating the vasodilatation mechanisms in humans. The major part of our understanding regarding the mechanisms of blood flow regulation is derived from studies using steady-state submaximal exercise.

In this study, we tested the effect of exhaustive whole body incremental exercise on venous plasma [ATP]. Comparison of previously reported data shows heterogeneity in physiological plasma [ATP] levels indicating that accurate estimation of this compound is currently difficult to perform. Also, ATP response during exercise remains controversial as plasma [ATP] has been reported to remain unchanged [22, 28, 29] or to increase rapidly at intensities above 60% of maximal workload [4]. In the present study, a continuous increase in venous plasma [ATP] from rest to maximal workload was observed. This pattern demonstrates that the rise in plasma [ATP] in response to exercise can be both large and rapid. Mortensen et al. demonstrated that ATP is released locally into both arterial and venous blood during exercise and that compression of the vasculature as well as hypoxia stimulates ATP release into plasma [19]. The present results provide evidence that ATP is released into venous plasma of contracting muscle and its concentration increases progressively with exercise intensity.

Determining plasma [ATP] is crucial for understanding its role in the skeletal muscle blood flow adjustments. ATP has been suggested to contribute to the local regulation of skeletal muscle blood flow by causing local vasodilatation [9]. It was previously shown that an arterial infusion of ATP in a human leg can cause vasodilatation similar to that observed during maximal exercise [4, 22]. Unfortunately, direct evidence for the role of ATP in blood flow regulation is lacking because of the absence of a selective receptor antagonist for human use [5]. An interesting observation is that both physical training [43] and chronic hypoxia [44] reduce the vasodilator response to arterially infused ATP, suggesting that purinergic P2 receptor sensitivity and/or ATP degradation in plasma is altered with training and hypoxia. Type of training can influence the physiological mechanisms of ATP release and its influence on muscle vessel dilatation during incremental exercise. We hypothesized that distinctive training types in highly trained sprinters, futsal players, and endurance athletes result in different responses of plasma [ATP]. Considerably higher plasma [ATP] in sprinters than in endurance athletes and futsal players, demonstrated during incremental treadmill test, indicates training-specific adaptations. Sprint training is mainly based on frequent stimulation in short time periods, contrary to endurance athletes whose training reflects the ability to maintain relatively low intensity workloads for longer periods of time. Futsal requires prolonged exercise characterized by frequent high-intensity efforts.

During exercise, skeletal muscle perfusion increases in direct proportion to the metabolic demand. Skeletal muscle blood flow can increase 100-fold from resting conditions up to 300–400 ml·min^−1^·kg^−1^ [45]. Several vasoactive compounds cooperate in synergy in regulation of skeletal muscle blood flow [15]. Skeletal muscles are one of the potential sources of extracellular ATP in venous blood. To explain the changes in plasma [ATP], we assumed that they are partially caused by total-body skeletal muscle mass. Sprinters, characterized by high SMM, obtained the highest absolute plasma [ATP] despite the lowest VO_2_max. Plasma [ATP] per kilogram of skeletal muscle mass eliminated differences between the groups of competitive athletes at rest, during maximal effort, and during recovery, which indirectly shows the influence of SMM in highly trained individuals. After normalizing ATP into 1 kilogram of skeletal muscle mass, differences between competitive athletes still exist during exercise, which suggests that not only skeletal muscle mass but also its specific adaptation to exhaustive exercise is important.

During exercise, the magnitude of ATP-induced vasodilatation may reflect increased demand for oxygen among athletes and healthy recreational runners. Speed-power training containing large proportion of anaerobic exercise results in adaptation to both rapid and intensified response of vessel dilatation. In contrast, endurance training, mainly based on aerobic exercise [46], seems to lead to moderate increase in ATP levels, spread over exercise time as their training is mainly focused on maintaining moderate intensities exercise for longer periods. In the control group, lower ATP_SMM_ suggests that training status and muscle adaptation to exercise are also crucial. In our opinion increased muscle capillarization in endurance athletes requires lower ATP increments than in speed-power athletes. Also, in speed-power athletes during exercise, increased hypoxia due to higher muscle mass requires an increase of vasodilatation to sustain exercise ability. Our finding about sport specialization impact appears to be in agreement with Laughlin & Roseguini [34] who demonstrated mode-specific training adaptive changes in vascular smooth muscle and endothelium as interactions of muscle fiber-type composition and muscle fiber recruitment patterns during exercise. However, the differences disappeared among our highly trained athletes if expressed per kilogram of SMM; thus we concluded that long-term intensive training of any kind leads to similar [ATP] response at rest and during recovery. Our results should be supported by further research.

Some substances have the ability to inhibit the local vasoconstrictor effect of increased sympathetic nerve activity. This phenomenon, termed functional sympatholysis, is also known to occur during exercise [22], but its importance involved in regulation is still under debate [47]. The ability of contracting skeletal muscle to blunt sympathetic vasoconstriction is critical for the proper regulation of tissue blood flow distribution and oxygen delivery. It allows increasing the skeletal muscle perfusion during high-intensity and/or large muscle mass exercise. “Functional sympatholysis” describes the ability of contracting skeletal muscle to block sympathetic vasoconstrictor activity. This is crucial to ensure proper blood flow distribution and O_2_ delivery to metabolically active skeletal muscle. The exact signalling mechanism responsible for sympatholysis in healthy humans is unknown, but its role varies between individuals, in particular differences in age and training status. Moreover, the compounds mediating functional sympatholysis in healthy humans are still unknown. It was proposed that ATP can play an important role by inducing local vasodilatation, overriding local sympathetic vasoconstriction, and stimulating the exercise pressor reflex [5].

To the best of our knowledge, the only vasoactive substance that has been shown to independently cause sympatholysis in humans is ATP. Local intra-arterial infusion of ATP showed a significant blunting of tyramine-induced vasoconstriction in the leg, similarly during moderate knee-extensor exercise [22]. ATP has a dose-dependent ability to attenuate sympathetic vasoconstriction during exercise and hypoxia. Low levels of ATP did not impact *α*_1_-mediated vasoconstriction while increasing ATP concentration gradually restricted *α*_1_-mediated vasoconstriction [48]. These observations are quite analogous to the intensity-dependent character of functional sympatholysis within contracting skeletal muscle [8]. One recent study provides evidence that *α*-adrenergic responsiveness, thus functional sympatholysis, can be improved by physical training [49]. With reference to what was indicated above, we assume that long-term training will lead to improvement in sympatholytic capacity. This assumption is consistent with our results as competitive athletes had significantly higher plasma [ATP] during exercise compared to healthy controls. Since ATP is capable of independently overriding sympathetic vasoconstriction in a dose-dependent manner, higher reported values at the end of exercise seem to be related to more efficient blood flow distribution to skeletal muscles. However, it is debatable whether the blood flow to exercising muscle is indeed constricted or only redistribution within muscle will be observed [50].

The balance between O_2_ delivery and its utilization during exercise depends on the specific needs of muscles which are influenced by the intensity, duration, and mode of exercise. Not only cardiac output increases during exercise but also blood flow is redistributed in such a way that over 80–90% of it can be directed to skeletal muscles [51]. The breakpoints observed during incremental exercise may reflect sudden changes in adjusting local oxygen demand according to its requirements; it is expected that oxygenation pattern changes might be reflected in whole body physiological responses [52]. The breakpoints of locomotor muscle oxygenation responses range between 75 and 90% of VO_2_max and are closely related to the traditionally determined threshold in pulmonary gas exchange, i.e., RCP [52]. There is currently a strong controversy as to whether these breakpoints in oxygenated and deoxygenated hemoglobin and myoglobin response in distinct regions of the body are corresponding to these thresholds in whole body responses and whether they can be used alternatively [52]. Nevertheless, erythrocytes are known to serve as oxygen sensors releasing vasoactive ATP in regions of low O_2_ tension. We suppose that low muscle oxygenation results in enhanced blood flow distribution to these regions to sustain muscle contractions. However, a further increase in exercise intensity causes an accumulation of metabolic by-products signalled by exercise-induced lactic acidosis and hyperventilation [53], a phenomenon known as RCP. We suggest that a rapid increase in plasma ATP curve is accompanied by RCP or even precedes this point. The main observation of our study was that highly trained athletes demonstrated remarkably increased plasma [ATP] during exercise compared to recreational participants. This may be caused by more optimal tissue oxygen delivery and vascularization of the muscle capillary bed. This study is the first attempt to prove that long-term training per se may result in considerable increases in plasma levels of the powerful vasoactive molecule ATP. Further research should focus on training-related plasma [ATP] alterations in the whole annual training cycle in relation to the predominant training type.

## 5. Conclusions

In summary, the obtained results show a significant impact of long-term training and sport specialization on plasma [ATP] at rest, during incremental treadmill exercise, and during maximal effort but not during the recovery period. The differences between athletic groups appear to be related to total-body skeletal muscle mass. A rapid increase in plasma [ATP] curve is concomitant with respiratory compensation point reflecting the partial inability to supply O_2_ to muscles during exercise.

## Figures and Tables

**Table 1:** Basic characteristics of the athletic groups and controls.

	Sprinters (n=11)	Futsal players (n=14)	Endurance athletes (n=16)	Control group (n=12)	ANOVA^*∗*^
Age (yr)	24.2±3.2	24.7±3.9	23.4±3.6	27.7±4.1^†^	0.03
Training experience (yr)	8.5±2.5	9.8±3.3	8.7±1.9	-	0.38
Height (cm)	186.2±4.6	181.7±5.6	181.8±6.1	180.4±5.6	0.09
Body mass (kg)	81.6±5.5^†^	77.0±6.7	73.4±7.2	76.7±7.7	0.03
BMI (kg/m^2^)	23.5±1.0	23.4±2.2	22.2±2.1	23.7±2.6	0.24
Total-body SMM (kg)	39.1±3.7	33.8±3.0^§^	32.4±3.2^a^	33.1±3.1^a^	<0.001
Total Tissue Fat (%)	12.56 ±2.22	17.46±2.67^§^	15.72 ±2.44	18.32 ±3.77^a^	<0.001
LA_rest_ (mmol·l^−1^)	1.4±0.6	1.4±0.4	1.2±0.3	1.4±0.3	0.36
LA_max_ (mmol·l^−1^)	11.0±1.4	11.4±2.0	11.6±1.9	10.1±1.1	0.15
HR_max_ (beats·min^−1^)	188±10	187±10	192±9	187±8	0.40
VO_2_max (ml·kg^−1^·min^−1^)	53.27±3.63^‡^	56.66±2.62^‡^	66.86±4.98	56.34±2.70^‡^	<0.001
VO_2_max (ml·kg SMM^−1^·min^−1^)	111.54±8.83^‡^	129.44±8.97^‡§^	151.65±13.49	130.46±7.04^‡a^	<0.001
RBC (10^12^·l^−1^)	5.11±0.29	4.91±0.25	4.81±0.43	4.92±0.14	0.12
Hb (mmol·l^−1^)	8.94±0.56	8.84±0.4	9.05±0.52	9.10±0.44	0.52
Hct (l·l^−1^)	0.42±0.03	0.42±0.02	0.42±0.03	0.42±0.02	0.59
MCV (fl)	83.16±2.19^†^	85.02±1.76	87.25±4.10	86.40±2.82	0.006
MCH (fmol)	1.75±0.04^‡^	1.80±0.04^†^	1.89±0.10	1.85±0.06^§^	<0.001
MCHC (mmol·l^−1^)	20.93±0.29	21.24±0.40	21.66±0.62	21.54±0.64	0.07
RDW (%)	11.67±0.69	11.70±0.40	11.58±0.58	11.51±0.52	0.82

^*∗*^One-way ANOVA. Values are means ± SD. ^†^*P* < 0.05, ^‡^*P* < 0.001, significantly different from endurance athletes, ^§^*P* < 0.01, ^a^*P* < 0.001, significantly different from sprinters. Abbreviations. BMI: body mass index; SMM: skeletal muscle mass; LA_rest_: resting lactate concentration; LA_max_: maximal lactate concentration; HR_max_: maximal heart rate; VO_2_max: maximal oxygen uptake; RBC: red blood cell; MCV: mean cell volume; MCH: mean cell hemoglobin; MCHC: mean corpuscular hemoglobin concentration; RDW: red blood cell distribution width.

## Data Availability

The raw data used to support the findings of this study are available from the corresponding author upon request.
